# PS-FW: A Hybrid Algorithm Based on Particle Swarm and Fireworks for Global Optimization

**DOI:** 10.1155/2018/6094685

**Published:** 2018-02-20

**Authors:** Shuangqing Chen, Yang Liu, Lixin Wei, Bing Guan

**Affiliations:** School of Petroleum Engineering, Northeast Petroleum University, Daqing 163318, China

## Abstract

Particle swarm optimization (PSO) and fireworks algorithm (FWA) are two recently developed optimization methods which have been applied in various areas due to their simplicity and efficiency. However, when being applied to high-dimensional optimization problems, PSO algorithm may be trapped in the local optima owing to the lack of powerful global exploration capability, and fireworks algorithm is difficult to converge in some cases because of its relatively low local exploitation efficiency for noncore fireworks. In this paper, a hybrid algorithm called PS-FW is presented, in which the modified operators of FWA are embedded into the solving process of PSO. In the iteration process, the abandonment and supplement mechanism is adopted to balance the exploration and exploitation ability of PS-FW, and the modified explosion operator and the novel mutation operator are proposed to speed up the global convergence and to avoid prematurity. To verify the performance of the proposed PS-FW algorithm, 22 high-dimensional benchmark functions have been employed, and it is compared with PSO, FWA, stdPSO, CPSO, CLPSO, FIPS, Frankenstein, and ALWPSO algorithms. Results show that the PS-FW algorithm is an efficient, robust, and fast converging optimization method for solving global optimization problems.

## 1. Introduction

Global optimization problems are common in engineering and other related fields [1–3], and it is usually difficult to solve the global optimization problems due to many local optima and complex search space, especially in high dimensions. For solving optimization problems, many methods have been reported in the past few years. Recently, the stochastic optimization algorithms have attracted increasing attention because they can get better solutions without any properties of the objective functions. Therefore, many effective metaheuristic algorithms have been presented, such as simulated annealing (SA) [4], differential evolution (DE) [5], genetic algorithm (GA) [6], particle swarm optimization (PSO) [7], ant colony optimization (ACO) [8], artificial bee colony (ABC) [9], and fireworks algorithm (FWA) [10].

Among these intelligent algorithms, the PSO and FWA have shown pretty outstanding performance in solving global optimization problems in the last several years. PSO algorithm is a population-based algorithm originally proposed by Kennedy and Eberhart [7], which is inspired by the foraging behavior of birds. Fireworks algorithm is a new swarm intelligence algorithm that is motivated by observing fireworks explosion. Owing to the less decision parameters, simple implementation, and good scalability, PSO and FWA have been widely applied since they were proposed, including shunting schedule optimization of electric multiple units depot [11], optimal operation of trunk natural gas pipelines [12], location optimization of logistics distribution center [13], artificial neural networks design [14], warehouse-scheduling [15], fertilization optimization [16], power system reconfiguration [17], and multimodal function optimization [18].

Although PSO and FWA are highly successful in solving some classes of global optimization problems, there are certain problems that need to be addressed when they are extended to handling complex high-dimensional optimization problems. The PSO algorithm has a significant efficiency in unimodal problems, but it can easily be trapped in local optima for multimodal problems. Moreover, the FWA is difficult to converge for the optimization problems which do not have their optimal solutions at the origin. This is because the two algorithms cannot keep the balance between the exploration and exploitation properly. Due to the optimal particle dominating the solving process, the PSO algorithm has inferior swarm diversity in the later stage of iterations and relatively poor exploration ability [19], while the fireworks and sparks in FWA are not well-informed by the whole swarm [20] and the FWA framework lacks the local search efficiency for noncore fireworks [21]. In order to improve the performance of PSO and FWA, a considerable number of modified algorithms have been proposed. For example, Nickabadi et al. presented AIWPSO algorithm, in which a new adaptive inertia weight approach was adopted [22]. By embedding a reverse predictor and adding a repulsive force into the basic algorithm, the RPPSO was developed [23]. Wang and Liu used three strategies to ameliorate the standard algorithm, including best neighbor replacement, abandoned mechanism, and chaotic searching [24]. Souravlias and Parsopoulos introduced a PSO-based variant, which could dynamically assign different computational budget for each particle based on the quality of its neighbor [25]. Based on self-adaption principle and bimodal Gaussian function, the advanced fireworks algorithm (AFWA) was proposed [26]. Liu et al. presented several methods for computing the explosion amplitude and number of sparks [27]. Pei et al. proposed to use the elite point of approximation landscape in the fireworks swarm and discussed the effectiveness of surrogate-assisted FWA [28]. Zheng et al. improved the new explosion operator, mutation operator, selection strategy, and mapping rules of FWA, which led to the formation of enhanced fireworks algorithm (EFWA) [29, 30] and dynamic search in fireworks algorithm (dynFWA) [31]. Zheng et al. proposed the new cooperative FWA framework (CoFFWA), in which the independent selection method and crowdedness-avoiding cooperative strategy were contained [21]. Li et al. investigated the operators of FWA and introduced a novel guiding spark in FWA [32] and proposed the adaptive fireworks algorithm (AFWA) [33] and bare bones fireworks algorithm (BBFWA) [34].

Hybrid algorithms can utilize various exploration and exploitation strategies for high-dimensional multimodal optimization problems, which have gradually become the new research areas. For example, Valdez et al. combined the advantages of PSO with GA and proposed a modified hybrid method [35]. In the new PS-ABC algorithm introduced by Li et al., the global optimum could be obtained by combining the local search phase in PSO with two global search phases in ABC [19]. Pandit et al. presented the SPSO-DE, in which the domain information of PSO and DE was shared with one another to overcome their respective weaknesses [36]. Through changing the generation and selection strategy of explosive spark, Gao and Diao proposed the CA-FWA [37]. Zhang et al. proposed BBO-FW algorithm which improved the interaction ability between fireworks [38]. By combining the FWA with the operators of DE, a novel hybrid optimization algorithm was proposed [20].

In this paper, by utilizing the exploitation ability of PSO and the exploration ability of FWA, a novel hybrid optimization algorithm called PS-FW is proposed. Based on the solving process of PSO algorithm, the operators of FWA are embedded into the update operation of the particle swarm. In the iteration process, in order to promote the balance of exploitation and exploration ability of PS-FW, we presented three major techniques. Firstly, the abandonment and supplement strategy is used, to abandon a certain number of particles with poor quality and to supplement the particle swarm with new individuals generated by FWA. Meanwhile, considering the information exchanges between the optimal firework and its neighbor in each dimension, the method for obtaining the explosion amplitude is designed as adaptive, and the mode of generating the explosion sparks is modified by combing the greedy algorithm. Furthermore, the conventional Gaussian mutation operator is abandoned, and the novel mutation operator based on the thought of the social cognition and learning is proposed. The performance of PS-FW is compared with several existing optimization algorithms. The experimental results show that the proposed PS-FW is more efficacious in solving the global optimization problems.

The rest of the paper is organized as follows: Section 2 describes the standard PSO and FWA.  Section 3 presents the PS-FW algorithm, in which the algorithm details are proposed.  Section 4 introduces the simulation results over 22 high-dimensional benchmark functions and the corresponding comparisons between PS-FW and other algorithms are executed. Finally, the conclusion is drawn in Section 5.

## 2. Related Work

### 2.1. PSO Algorithm

In PSO algorithm, the particles scatter in search space of the optimization problems and each particle denotes a feasible solution. Each particle contains three aspects of information: the current position *x*_*i*_, the velocity *v*_*i*_, and the previous best position *pbest*_*i*_. Assume that the optimization problem is *D*-dimensional and *M* represents the size of the swarm population; then the position and velocity of *i*th (*i* = 1,2,…, *M*) particle can be denoted as *x*_*i*_ = (*x*_*i*,1_, *x*_*i*,2_,…, *x*_*i*,*D*_) and *v*_*i*_ = (*v*_*i*,1_, *v*_*i*,2_,…, *v*_*i*,*D*_), respectively, while the previous best position is represented as *pbest*_*i*_ = (*pbest*_*i*,1_, *pbest*_*i*,2_,…, *pbest*_*i*,*D*_). Besides, the best position encountered by the entire particles so far is known as current global best position *gbest*_*i*_ = (*gbest*_1_, *gbest*_2_,…, *gbest*_*D*_). In each generation, *v*_*i*_ and *x*_*i*_ are updated by the following equations:

(1) vi,kt+1=w·vi,kt+c1·r1·pbesti,kt−xi,kt+c2·r2·gbestt−xi,kt,

(2) xi,kt+1=xi,kt+vi,kt+1,

where *c*_1_ and *c*_2_ are two learning factors that indicate the influence of the cognitive and social components, *r*_1_ and *r*_2_ are the random real numbers in interval [0, 1], respectively, and *w* is the inertia weight which controls the convergence speed of the algorithm.

### 2.2. Fireworks Algorithm

In FWA, a firework or a spark denotes a potential solution of optimization problems, while the process of producing sparks from fireworks represents a search in the feasible space. As in other optimization algorithms, the optimal solutions are obtained by successive iterations. In each iteration, the sparks can be produced by two ways: the explosion and the Gaussian mutation. The explosion of fireworks is dominated by the explosion amplitude and the number of explosion sparks. Compared to the fireworks with lower fitness, the fireworks with better fitness will have smaller explosion amplitude and more explosion sparks. Suppose that *N* denotes the number of fireworks; then the *i*th (*i* = 1,2,…, *N*) firework can be denoted as $\bar{x}=({\bar{x}}_{i,1},{\bar{x}}_{i,2},\ldots ,{\bar{x}}_{i,D})$ for *D*-dimensional optimization problems. Besides, the explosion amplitude can be obtained by (3) and the sparks number can be calculated by (4):

(3) Ai=A^·fx¯i−ymin+ε∑i=1Nfx¯i−ymin+ε,

(4) si=Me·ymax−fx¯i+ε∑i=1Nymax−fx¯i+ε,

where $f(\bar{x})$ denotes the objective function value of the *i*th firework, *i* = 1,2,…, *N*, *A*_*i*_ and *s*_*i*_ are the explosion amplitude and the number of explosion sparks of the *i*th firework, respectively, $y_{max}=\max\left(f({\bar{x}}_{i})\right)$, $y_{min}=\min\left(f({\bar{x}}_{i})\right)$, $\hat{A}$ and *M*_*e*_ are two constants that dominate the explosion amplitude and the number of explosion sparks, respectively, and *ε* is the machine epsilon.

Moreover, the bounds of *s*_*i*_ are defined as follows:

(5) $$\begin{array}{c} s_{i}=\left\{\begin{array}{cc} round\left(a\cdot M_{e}\right), & s_{i}<a\cdot M_{e}\\ round\left(b\cdot M_{e}\right), & s_{i}>b\cdot M_{e}\\ round\left(s_{i}\right), & otherwise, \end{array}\right. \end{array}$$

where *a*, *b* are two constants that control the minimum and maximum of population size, respectively.

In order to generate each explosion spark of *i*th firework, an offset is added to ${\bar{x}}_{i}$ according to the following equation:

(6) $$\begin{array}{c} {\hat{x}}_{i}^{j}={\bar{x}}_{i}+\Delta h, \end{array}$$

where ${\hat{x}}_{i}^{j}$ is the *j*th explosion spark of *i*th firework and $\Delta h=A_{i}\cdot rand(-1,1)\cdot \hat{B}$, where $\hat{B}$ is a *D*-dimensional vector which has ${\hat{z}}_{i}^{j}$ values of 1 and $D-{\hat{z}}_{i}^{j}$ values of 0, where ${\hat{z}}_{i}^{j}$ denotes the number of randomly selected dimensions of $\bar{x}$ and ${\hat{z}}_{i}^{j}=D\cdot rand()$, *j* = 1,2,…, *s*_*i*_, where rand(−1,1) and rand() are random numbers in the intervals [−1, 1] and [0, 1], respectively.

Another type of sparks known as the Gaussian sparks is generated based on the Gaussian mutation operator. In each generation, a certain number of Gaussian sparks are generated and each Gaussian spark is transformed from a firework which is selected randomly. For the selected firework ${\bar{x}}_{i}$, its Gaussian spark is generated based on

(7) $$\begin{array}{c} {\tilde{x}}_{j}=\left(\;\tilde{O}-{\tilde{B}}_{i}\;\right)\cdot {\bar{x}}_{i}+Gaussian\left(\;1,1\;\right)\cdot {\bar{x}}_{i}\cdot {\tilde{B}}_{i}, \end{array}$$

where ${\tilde{x}}_{j}$ is the *j*th Gaussian spark, $\tilde{O}$ is a *D*-dimensional vector whose values are 1 in each dimension, $\tilde{B}$ is a *D*-dimensional vector which has ${\tilde{z}}_{i}$ values of 1 and $D-{\tilde{z}}_{i}$ values of 0, ${\tilde{z}}_{i}$ represents the number of randomly selected dimensions of ${\bar{x}}_{i}$ and ${\tilde{z}}_{i}=D\cdot rand()$, and Gaussian(1, −1) represents a random number subordinated to the Gaussian distribution with the mean of 1 and the standard deviation of 1.

For the purpose of passing information to the next generation, new fireworks populations are chosen to continue the iteration. All the fireworks, the explosion sparks, and Gaussian sparks have the chance to be selected for the next iteration. The location with best fitness is kept for the next generation, while the other *N* − 1 locations are selected based on the selection operator and the selection operator is denoted as follows:

(8) RXi=∑j∈KdXi,Xj=∑j∈KXi−Xj,pXi=RXi∑k∈KRXk,

where *K* denotes the set comprised of all the original fireworks and both types of sparks, *X*_*i*_, *X*_*j*_, and *X*_*k*_ are *i*th, *j*th, and *k*th location of *K*, respectively, *R*(*X*_*i*_) is the distance between *i*th location and the rest of all the locations, and *p*(*X*_*i*_) denotes the probability of being selected for the *i*th location.

## 3. Hybrid Optimization Algorithm Based on PSO and FWA

The exploitation process focuses on utilizing the existing information to look for better solutions, whereas the exploration process attaches importance to seek the optimal solutions in the entire space. For PSO, under the guidance of their historical best solutions and the current global best solution, the particles can quickly find better solutions, and the excellent exploitation efficiency of algorithm is shown. In FWA, the fireworks can find the global optimal solution in the whole search space by performing explosion and mutation operations while the outstanding exploration capability of FWA is demonstrated. To utilize the advantages of the two algorithms, a hybrid optimization method (PS-FW) based on PSO and FWA is proposed.

### 3.1. Feasibility Analysis

**Figure 1:**
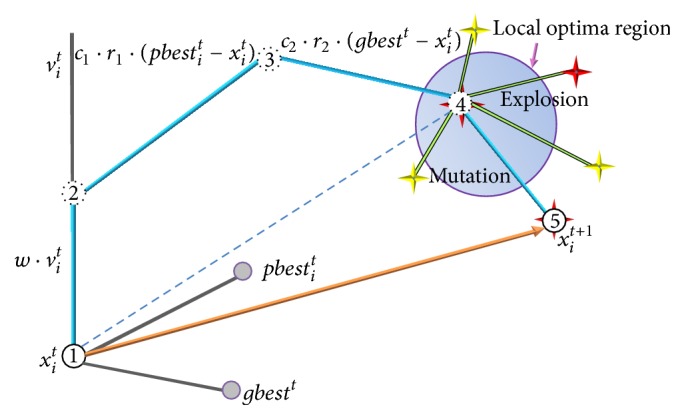
Optimization mechanism of adding operators of FWA to PSO algorithm.

**Figure 2:**
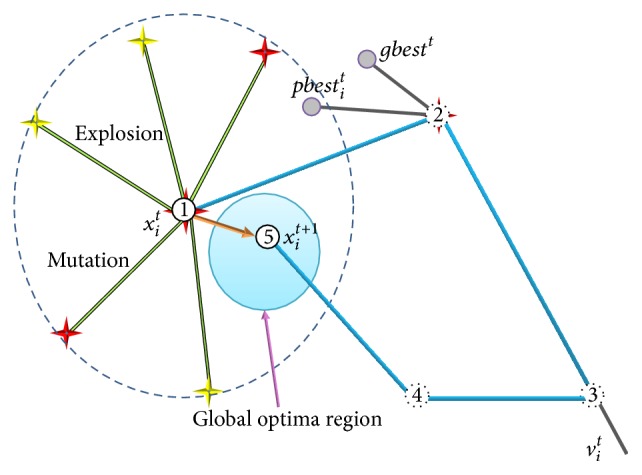
Optimization mechanism of adding operators of PSO to FWA.

The formation of a hybrid algorithm is mainly due to the effective combination of the operators of its composition algorithms in a certain way. To clarify the performance enhancement caused by combining the PSO algorithm with fireworks algorithm, we draw Figures 1 and 2 to illustrate the optimization mechanism. As shown in Figure 1, for standard PSO algorithm, the *i*th particle moves from point 1 to point 4 under the common influence of velocity inertia, self-cognition, and social information. When the operators of FWA are added, the particle is transformed into firework and performs explosion and mutation operations and eventually reaches the position of firework or sparks, such as point 5 shown in Figure 1. By performing the operators of FWA, the particle can explore better solutions in multiple directions and jump out of the local optima region as depicted in Figure 1. Thus we can argue that the operators of FWA improve the global search ability of PSO algorithm. As we know, the searching region is determined by the explosion amplitude and fireworks with poor quality have bigger amplitude, which may lead to an uncomprehensive search without considering the cooperation with other fireworks. When the firework with poor quality generates the explosion sparks and mutation sparks, the new selected location may skip over the global optima region without the attraction from the rest of fireworks and arrive at point 2. By adding the operators of PSO after the *i*th firework updates its location, the information of its own historical best location and current global best location is taken into account; then the new solution is found in point 5, which is shown in Figure 2. Therefore, the operators of PSO could strengthen the local search efficiency of FWA. Based on the above analysis, it is concluded that the combination of PSO and FWA is an effective way to form a superior optimization algorithm.

### 3.2. The Abandonment and Supplement Mechanism

The particles with their memory ability can be quickly converged to the current optimal solution. However, the aggregation effect of the particle swarm reduces the diversity of the population, which makes the search in the whole feasible space inefficient. In this paper, in order to enhance the balance between exploitation ability and exploration ability of PS-FW, we adopt the abandonment and supplement strategy which includes three main steps. (i) All the particles in the particle swarm *x*_1_, *x*_2_,…, *x*_*M*_ are sorted in ascending order. Then the *P*_num_ particles with better fitness are retained for the next iteration, and the FW_num_ (satisfying *P*_num_ + FW_num_ = *M*) particles with lower fitness are abandoned. (ii) The *P*_num_ excellent individuals denoted as *x*_*F*1_, *x*_*F*2_,…, *x*_*FP*_num__ are used to implement the explosion operator, the mutation operator, and the selection operator. (iii) The new individuals obtained by the operators of FWA are added to the original population, to balance the number of particles and to generate the new particle swarm for the next iteration. The abandonment and supplement strategy not only retains the information of the excellent individuals so that they can participate in the subsequent calculation, but also avoids the individuals with poor quality wasting computing resources. However, the problem arises: how to determine *P*_num_? For this, through analyzing the process of solving the optimization problems, we should enhance the exploration ability of the algorithm and search the optimal solution in the global scope at early stage of iterations, which means the number of particles executing the operators of FWA should be the majority. In the later stage of iteration, we should focus on searching around the current global optimal solution, so the number of excellent individuals retained in the algorithm should be more. Based on the discussion above, the calculation of FW_num_ in this paper is shown in (9), in which FW_num_ decreases with iteration process.

(9) FWnum=roundFWmax−FWmin·Imax−tImaxr+FWmin,

where FW_max_ and FW_min_ are the upper and lower bounds of number of abandoned particles, respectively, *I*_max_ is the maximum number of iterations, *t* denotes the current number of iterations, round[] indicates that the values in brackets are rounded, and *r* represents a positive integer.

### 3.3. Modified Explosion Operator

#### 3.3.1. Adaptive Explosion Amplitude

Based on the analysis above, the definition of the explosion amplitude in standard FWA limits the diversity of the explosion sparks generated by the excellent fireworks, thus decreasing the local search ability of algorithm. In the enhanced fireworks algorithm (EFWA) [29], in order to avoid the weakness of the explosion amplitude generation in FWA, a minimal explosion amplitude check mechanism is proposed, which defines the explosion amplitude less than a certain threshold to obtain the same value as the threshold while the threshold is reducing with the iteration process. Suppose that *δ* denotes the threshold of explosion amplitude; then the explosion amplitude less than the threshold is defined as (10) in EFWA.

(10) $$\begin{array}{c} \hat{A}={\hat{A}}_{init}-\frac{{\hat{A}}_{init}-{\hat{A}}_{final}}{I_{max}}\cdot \sqrt{\left(2I_{max}-t\right)t}, \end{array}$$

where ${\hat{A}}_{init}$ and ${\hat{A}}_{final}$ are the upper and lower bounds of the explosion amplitude, respectively.

In this paper, based on the minimal explosion amplitude detection mechanism, the basic explosion amplitude of each firework is calculated according to (3), and the explosion amplitude is adjusted by the following two methods.

(1) For the fireworks whose explosion amplitude is greater than the threshold *δ*, a control factor *λ* of the explosion amplitude is added. The control factor makes the explosion sparks generated by the algorithm have larger search scope in the early stage of iterations, which can effectively enhance the exploration ability of the algorithm. In the later stage of iterations, the explosion amplitude is reduced to improve the search efficiency around the current global optimal solution. The adjustment of the explosion amplitude is shown in (11), and the control factor is calculated as shown in (12).

(11) A^i=A^i·λ,∀A^i>δ,

(12) λ=λmin·λmaxλmin1/1+t/Imax,

where *λ*_max_ and *λ*_min_ are the lower and upper bounds of the control factor, respectively.

(2) When the explosion amplitude of firework ${\bar{x}}_{i}$ is less than the threshold, the optimal firework and its neighbor information are used to determine the explosion amplitude in the hybrid algorithm. Since the PS-FW algorithm is based on the framework of PSO, the position of all individuals will approach the current best position, which leads to the fitness of current optimal individual close to its neighbor individuals. That is to say, if the explosion amplitude of a firework is too small, indicating that the firework may be located near the current best location, therefore, by considering the deviation information of all corresponding dimensions between the current best firework and its neighbor firework, a new explosion amplitude of the firework ${\bar{x}}_{i}$ is generated. The explosion amplitude generation method can adaptively optimize the solving process, which can be interpreted from two aspects. When the algorithm is in the early iteration stage, the position of fireworks is scattered, and the deviation in dimensions between the optimal firework and its neighbor firework is larger, which leads to the larger explosion amplitude and the improved probability of finding the global optimal solution. As the algorithm enters the later iterations, the fireworks gather around the current best location, and the offset of each dimension between the current best firework and its neighbor firework is reduced, which results in the decrement of explosion amplitude and the improvement of the local search ability for PS-FW. There are two main steps to obtain the explosion amplitude. (i) Randomly select a firework ${\bar{x}}_{j}$ around the current optimal firework according to the fitness. (ii) Update the explosion amplitude of the *i*th firework according to the following equation:

(13) $$\begin{array}{c} {\hat{A}}_{i}=\frac{\sum_{k=1}^{D}\left(\left|{\bar{x}}_{best,k}-{\bar{x}}_{j,k}\right|\right)}{D}, \end{array}$$

where ${\bar{x}}_{best,k}$ denotes the value of the *k*th dimension of current optimal firework.

#### 3.3.2. Modified Explosion Sparks Generation

In FWA, when generating an explosion spark, the offset Δ*h* is only calculated once, which results in the same changes for all the selected dimensions and an ineffective search for different directions. In the PS-FW algorithm proposed in this paper, a new explosion sparks generation method is introduced. Firstly, when generating the explosion sparks, the location offset is performed in all the dimensions of the fireworks instead of randomly selecting part of dimensions. Furthermore, for each dimension of the fireworks, the different offsets are calculated according to (14), thereby increasing the diversity of the explosion sparks and the global search capability of the hybrid algorithm. Meanwhile, suppose that ${\bar{x}}_{temp}$ denotes the *i*th firework without a location offset and ${\bar{x}}_{+}$ indicates the *i*th firework whose *k*th dimension adds a offset; then ${\bar{x}}_{-}$ denotes the *i*th firework whose *k*th dimension subtracts an offset. As shown in (15), inspired by greedy algorithm, when the fireworks generate their explosion sparks, the hybrid algorithm determines which offset to be selected based on the value of objective function, which can effectively improve the local search capability of the algorithm and accelerate the convergence.

(14) Δh^k=A^·Gaussian0,1,

(15) x^i,kj=x¯i,k+Δh^k,fx¯+≤min⁡fx¯temp,fx¯−x¯i,k−Δh^k,fx¯−≤min⁡fx¯temp,fx¯+x¯i,k,fx¯temp≤min⁡fx¯+,fx¯−,

where ${\hat{x}}_{i,k}^{j}$ and $\Delta {\hat{h}}_{k}$ are the value and offset of the *k*th dimension of the *j*th explosion spark for the *i*th firework, respectively, Gaussian(0,1) represents a random number that follows the standard normal distribution, *i* and *j* are integers in the intervals [1, *P*_num_] and [1, *s*_*i*_], respectively, and min() indicates the minimum values in parentheses.

Assume that num_*E*_ denotes the total number of explosion sparks generated by all fireworks, *S*_min_ and *S*_max_ represent the lower and upper bounds for the search scope, and *S*_min,*k*_ and *S*_max,*k*_ are corresponding to the bounds of *k*th dimension, respectively. Based on the explosion operator introduced in Sections 3.3.1 and 3.3.2, the detailed codes of explosion operator are represented in Algorithm 1.

### 3.4. Novel Mutation Operator

As the Gaussian mutation operator effectively increases the diversity of feasible solutions, the performance of traditional FWA has been significantly improved. However, the numerical experiments show that the combined application of Gaussian operator and mapping operator makes the Gaussian sparks mostly concentrated around the zero point, which is the reason why FWA has the fast convergence speed for the problems with their optimal solutions at zero [31]. In order to improve the adaptability of the algorithm for the nonzero optimization problems and maintain the contribution of the mutation operator to the population diversity, a new mutation operator is proposed in the PS-FW. Compared with the standard FWA, there are two main differences in this paper. (i) In PS-FW, we randomly select a certain number of explosion sparks to generate the mutation sparks instead of using the fireworks. Because the explosion sparks have better quality compared to the fireworks based on (15), the mutation sparks generated by the explosion sparks can effectively enrich the diversity of the population and have better global search ability. (ii) In this paper, the Gaussian random number is no longer used in mutation operator and the interaction mechanism of particles in PSO is used for reference to design the mutation operator. The mutation sparks generated by our mutation operator can not only maintain the better information of the explosion sparks, but also have a proper movement towards the current best location, which leads to promoting the convergence of hybrid algorithm. The proposed mutation operator is shown as follows.

(16) $$\begin{array}{c} {\tilde{x}}_{i,k}=\mu_{1}\cdot \left(\;{\hat{x}}_{best,k}-{\hat{x}}_{j,k}\;\right)+\mu_{2}\cdot {\hat{x}}_{j,k}, \end{array}$$

where ${\tilde{x}}_{i,k}$ and ${\hat{x}}_{j,k}$ indicate the value of *k*th dimension of *i*th mutation spark and *j*th explosion spark, respectively, ${\hat{x}}_{best,k}$ is the current optimal explosion spark, *μ*_1_ and *μ*_2_ are the random number in [0, 1], and *j* denotes the random integer of the interval [1, num_*E*_], *i* = 1,2,…, num_*M*_, where num_*M*_ indicates the total number of mutation sparks.

The detailed codes of mutation operator are represented in Algorithm 2.

### 3.5. Main Process of PS-FW

In PS-FW, the algorithm consists of two main stages, which are initialization stage and iterations stage. In the initialization phase, we need to initialize the position and velocity of the particle swarm, as well as to initialize the control parameters. In the iterative phase, the PS-FW algorithm inherits all the parameters and operators of the PSO algorithm, and all particles are used as the main carrier for storing feasible solutions. Firstly, in each iteration, the particles update their speed and position according to the operators of the PSO algorithm and then perform the abandonment and supplement operation. Besides, in the process of generating the supplement particles by using the operators of FWA, we first generate num_*E*_ explosion sparks according to the excellent *P*_num_ particles and the modified explosion operator; then the fitness of the explosion sparks is given. Secondly, the num_*M*_ mutation sparks are generated by the explosion sparks and the novel mutation operator. Finally, the FW_num_ supplement individuals are selected by the combination of elite strategy and roulette strategy. When each iteration is completed, it is judged whether the termination condition is satisfied. If the stopping criterion is matched, the iteration will be stopped and the best solutions are output. Otherwise, the iteration phase will be repeated.

In the procedures above, there are two points to be noted. (i) In the implementation process of the hybrid algorithm, it is necessary to detect whether the position of individuals is within the feasible scope while the individuals consist of particles, fireworks, explosion sparks, and mutation sparks. As shown in (17), if the position of individuals exceeds the feasible scope, it is adjusted by using the mapping criteria in the EFWA algorithm [29].

(17) Yi,k=Smin,k+e·Smax,k−Smin,k∀Yi,k>Smax,k or Yi,k<Smin,k,

where *Y*_*i*,*k*_ indicates the value of the *k*th dimension of the individual and *e* is a random number in [0, 1].

(ii) The selection strategy of FWA based on the density of feasible solutions is abandoned in the PS-FW algorithm. Although it is possible to maintain the diversity of the population by selecting the location which has fewer individuals around with a larger probability, relatively more time is wasted by calculating the spatial distance between the individuals and the efficiency of the algorithm is reduced. Therefore, a selection strategy based on fitness is applied in PS-FW, which means the elite strategy is used to retain the best individual directly into the next iteration and the remaining FW_num_ − 1 locations are selected by the roulette criterion according to the fitness.

According to the description above, the main codes of the PS-FW algorithm are given in Algorithm 3.

## 4. Problems, Experiments, and Discussion

### 4.1. Test Problems

In order to evaluate the efficacy and accuracy of the proposed algorithm, the performance of PS-FW is tested by the 22 high-dimensional benchmark functions. The test problems which consist of multimodal functions and unimodal functions are listed in Table 1, and the corresponding optimal solutions and search scope are presented in Table 1. Compared with solving unimodal problems, it is difficult to find the global optimum of multimodal problems because the local optima will induce the optimization algorithms' fall into their surroundings. Therefore, if the algorithm can efficiently find the optimal solutions of multimodal functions, it can be proved that the algorithm is an excellent optimization algorithm.

### 4.2. Comparison of PS-FW with PSO and FWA

In this section, we compare the performance of the PS-FW with the PSO and FWA based on the 22 benchmark functions. In order to explore global optimization capability of the three algorithms on solving the high-dimensional optimization problem, three experiments with different dimensions are carried out. The dimensions of experiments are set to *D* = 30, *D* = 60, and *D* = 100, respectively, and each algorithm is used to solve all the benchmark functions 20 times independently. In order to make a fair comparison, the general control parameters of algorithms such as the maximum number of iterations (*I*_max_) and the population size (*M*) are set to be of the same value. *I*_max_ is set to 1000 and *M* is set to 50 for each function. Besides, the algorithms used in the experiment are coded by MATLAB 14.0, and the experiment platform is a personal computer with Core i5, 2.02 GHz CPU, 4 G memory, and Windows 7. For the purpose of eliminating the impact on performance caused by the difference in parameter settings, the main control parameters of PS-FW algorithm are consistent with those of PSO and FWA, and the other detailed control parameters are shown in Table 2.

**Figure 3:**
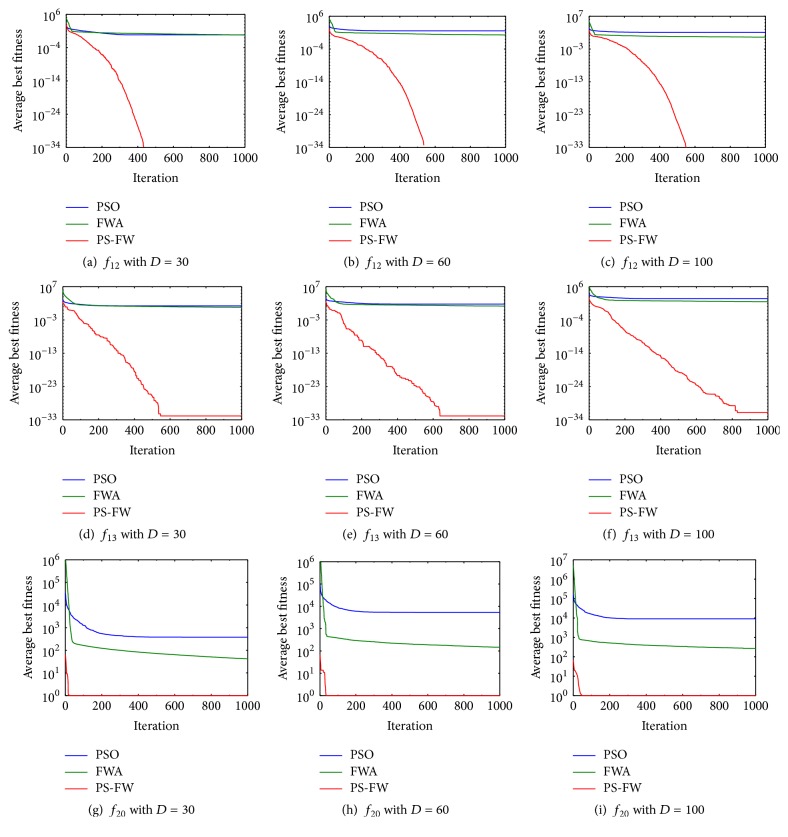
Convergence curves of PSO, FWA, and PS-FW for functions *f*_12_, *f*_13_, and *f*_20_.

For all the benchmark functions, the mean and standard deviation of best solutions obtained by PS-FW and other algorithms in 20 independent runs are recorded, and the optimization results are shown in Tables 3–5. Meanwhile, the ranks are also presented in tables, and the three algorithms are ranked mainly based on the mean of best solutions. In addition, the average convergence speed of the proposed PS-FW is compared with other algorithms for functions *f*_12_, *f*_13_, and *f*_20_; therefore the convergence curves are shown in Figure 3.

According to the ranks shown in Tables 3–5, the average values of best solutions for the proposed PS-FW outperform those of the other algorithms. Besides, the performance of PS-FW over standard deviation of best solutions is also better than the rest of the algorithms. For 22 problems with *D* = 30, the PS-FW can obtain the global optimum of *f*_2_, *f*_3_, *f*_4_, *f*_5_, *f*_6_, *f*_8_, *f*_12_, *f*_15_, *f*_17_, *f*_18_, *f*_20_, and *f*_21_, which shows excellent ability for solving optimization problems. As the dimensions of problems increase, the hybrid algorithm maintains outstanding performance and obtains the optimal solutions of the 10 functions, except for functions *f*_3_ and *f*_6_, compared with results in Table 3. When the dimensions of problems are 60 and 100, PS-FW can get the global optimum of functions *f*_3_ and *f*_6_, but not each run can succeed. This is because functions *f*_3_ and *f*_6_ are multimodal problems and the number of local optima increases rapidly as the dimensions of the problems increase, which adds the difficulty of avoiding trapping in the local optima. In addition, according to the ranks and values shown in Tables 3–5, the PS-FW can get the highest rank for all the functions. It is also needed to point out that the PS-FW obtains more stable solutions than PSO and FWA for all problems with the increasing of dimensionality. The convergence speed of the three algorithms can be seen in Figure 3, and the descend rate of average best solutions of PS-FW is obviously higher than the other two algorithms. This is because the advantages of PSO and FWA are combined into the PS-FW so that the hybrid algorithm enhances its global and local search ability. Therefore, PS-FW is efficient and robust in dealing with the high-dimensional benchmark functions.

**Figure 4:**
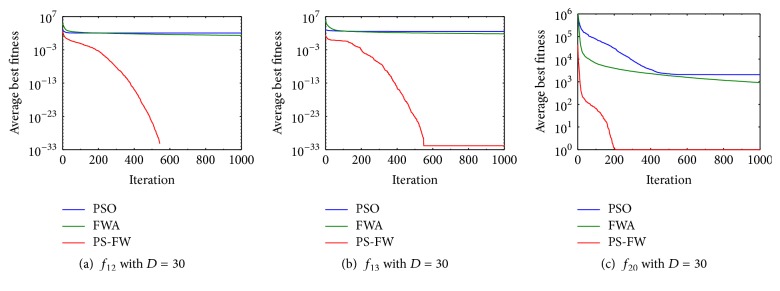
Convergence curves of PSO, FWA, and PS-FW for functions *f*_12_, *f*_13_, and *f*_20_.

From the above analysis, it is possible to show that the PS-FW algorithm performs well in solving the functions in Table 1. However, because the optimums of these functions are mostly at the origin, we need to further explore the performance of PS-FW algorithm on the nonzero problems. Then the experiment of nonzero problems is carried out to prove the comprehensive performance of PS-FW. In this experiment, the optimums of test functions derived from Table 1 are shifted and the specific values are displayed in Table 6. In addition, in order to achieve a fair comparison between the experiments, the parameters settings of three algorithms are consistent with Table 2 and the dimension is set to *D* = 30. The optimization results of three algorithms are shown in Table 7 and the convergence curves of three algorithms over functions *f*_12_, *f*_13_, and *f*_20_ are displayed in Figure 4.

From Table 7, we can know that the PS-FW algorithm keeps high performance and can obtain the optimal solutions of 11 functions in Table 6. Besides, the PS-FW achieves the best rank of three algorithms for all the functions with shift optimums, which present the powerful solving ability over optimization problems with nonzero optimums. By comparing Table 7 with Table 3, it is known that fireworks algorithm is relatively weak in searching for nonzero optimums. However, the PS-FW algorithm that derives from the fireworks algorithm and covers operators of PSO shows better performance, which demonstrates the correctness of the combination of the two algorithms. In addition, the result of PS-FW over function 16 is worse than the previous experiment. This is because *f*_16_ is a multimodal function and the slight deviations from the optimums can cause the significant increase in the value of the objective function. By observing the convergence curves in Figure 4, we can state that the convergence speed of the PS-FW also remains fast. In order to determine whether the convergence performance of PS-FW algorithm is superior to the other two algorithms more clearly, we compute the number of successful runs (success rate) and the average number of iterations in successful runs for each function in Table 6. The optimal solutions obtained by different algorithms are various, so we define the convergence criterion for each function. The convergence criterion can be introduced as that if the best solutions *f*_find_ found by each of algorithms are satisfying (18) in a run [39], the run is considered to be successful, and the minimum number of iterations satisfying the convergence criterion is counted to calculate the average number of iterations.

(18) $$\begin{array}{c} \left|\;f_{find}-f_{opti}\;\right|<\tau , \end{array}$$

where *f*_opti_ is the optimum of function and *τ* denotes the error of algorithm.

Suppose that ST denotes the number of successful runs, AI indicates the average number of iterations in successful runs, and *U* denotes the iterations number when there are no successful runs after 20 runs and its value is set to greater than *I*_max_; then Table 8 is shown as follows.

According to the statistical results and ranks presented in Table 8, the success rate and the average iterations number of PS-FW in 20 runs are both superior to other algorithms. For all the benchmark functions in Table 6, the proposed PS-FW can satisfy the convergence criterion for all the 20 runs, whereas the other algorithms can only converge to the criterion for several functions. In addition, the PS-FW obtains the highest ranks for the average number of iterations in successful runs and can converge to the criterion by a relatively small number of iterations. In summary, the PS-FW outperforms the other algorithms in terms of stability and convergence speed and is an efficacious algorithm for optimization problems whose optimums are at origin or are shifted.

### 4.3. Comparison of PS-FW with PSO Variants

In this section, we compare the performance of the proposed PS-FW with several existing variants of PSO which are introduced in a published paper. The comparison is based on the 12 benchmark functions introduced in the paper of Nickabadi et al. [22] and the orders of functions are consistent with that in this paper. In order to make a fair comparison, the run times and maximum iterations of PS-FW are set to 30 and 200,000, respectively, and the other parameters are set to be the same as those in Section 4.2. The dimension of test problems is set to *D* = 30, and the mean and standard deviation of best solutions obtained by algorithms are calculated. The contrast results are presented in Table 9, and the rank of each algorithm is counted and shown.

According to the results of Table 9, the PS-FW outperforms the other six PSO variants on both the average values and standard deviation of best solutions after 200,000 iterations. Among the 12 benchmark functions, the PS-FW can obtain the optimum of 10 functions, which manifests the highly powerful ability to find the global optimal solution. In addition, the PS-FW acquires the highest rank over almost all the test problems except the function *f*_11_, which indicates the PS-FW has significant improvement than other algorithms. Besides the analysis of numerical results obtained by PS-FW and other algorithms, we applied the nonparametric statistical tests to prove the superiority of the PS-FW. The Friedman test and Bonferroni-Dunn test are adopted to compare the performance of PS-FW with the other algorithms.

The Friedman test is a multiple comparison test, to detect the significant differences among algorithms based on the sets of data [40]. The algorithms are ranked in Friedman test, which means the algorithm with the best performance is ranked minimum, the worst gets the maximum rank, and so on. In this section, the mean and standard deviation of best solutions based on Table 9 are conducted with the Friedman test; therefore the results are given in Table 10. Through observing the results of Friedman test in Table 10, all the *p* value are lower than the level of significance considered *α* = 0.01, which indicates that the significant differences among the seven algorithms do exist. According to the ranks obtained by the Friedman test in Table 10, the PS-FW has the best performance on the mean and standard deviation of best solutions followed by ALWPSO, CLPSO, and the other four algorithms. Therefore, we can conclude that the accuracy of solutions obtained by PS-FW is better than other algorithms. However, the Friedman test can only detect whether there are significant differences among all the algorithms, but is unable to conduct the proper comparisons between PS-FW and each of the other algorithms. Hence the Bonferroni-Dunn test is executed to check the superiority of PS-FW.

The Bonferroni-Dunn test can be very intuitive to detect the significant difference between the two or more algorithms. For Bonferroni-Dunn test, the judgment condition for the existence of significant difference between the two algorithms is that their mean ranks differ by at least the critical difference (CD), and the equation of calculating the critical difference is as follows [41]:

(19) $$\begin{array}{c} {CD}_{\alpha }=q_{\alpha }\sqrt{\frac{N_{i}\left(N_{i}+1\right)}{6N_{f}}}, \end{array}$$

where *N*_*i*_ and *N*_*f*_ are the number of algorithms and benchmark functions and the critical values *q*_*α*_ at the probability level *a* are presented as follows:

(20) q0.05=2.77,q0.1=2.54.

By utilizing (19) and (20), the critical difference is shown as follows:

(21) CD0.05=2.44,CD0.1=2.24.

**Figure 5:**
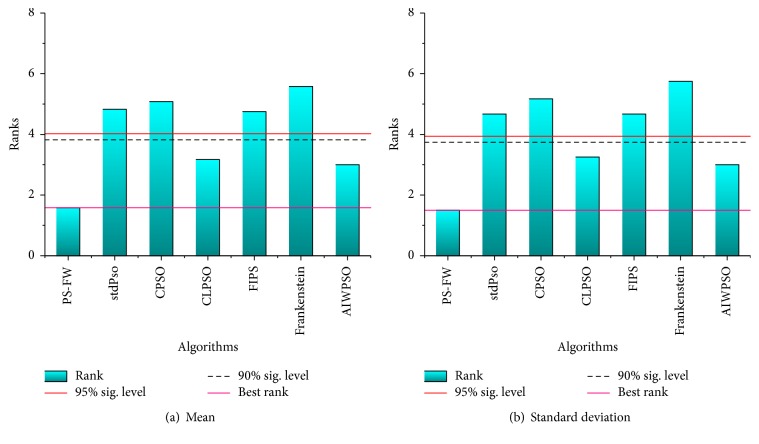
The bar chart of Bonferroni-Dunn test for PS-FW and other PSO variants over mean and standard deviation of best solutions based on Table 10.

Here we carry out the Bonferroni-Dunn test for the mean of best solutions, success rate, and average number of iterations of successful runs on the basis of the ranks obtained by the Friedman test. In order to provide a more intuitive display of the results obtained by Bonferroni-Dunn test, we illustrate the critical differences among the seven algorithms in Figure 5. For the purpose of comparing the algorithms clearly, a horizontal line which indicates the threshold for the best performing algorithm (the one with pink color) is drawn in the graphs. In addition, another two lines which represent each level of significance considered in the paper are also drawn, and their heights are equal to the sum of minimum rank and the corresponding CD. Then if the bars exceed the lines of significant level, the corresponding algorithms are proved to have worse performance than the best performing algorithm. By observing the results of Bonferroni-Dunn test in Figure 5(a), the bar of the PS-FW has the lowest height among all the algorithms, and the heights of bars corresponding to the stdPSO, CPSO, FIPS, and Frankenstein exceed the lines of significant level, which indicates that the PS-FW performs significantly better than these four algorithms over the solutions accuracy. In addition, the PS-FW acquires the best rank over the standard deviation according to Figure 5(b), and the PS-FW has the obvious advantage compared to the stdPSO, CPSO, FIPS, and Frankenstein. Therefore, we can conclude that the PS-FW is the best performing algorithm followed by ALWPSO, CLPSO, and other four algorithms, and the advantages of PS-FW on the efficiency and solutions accuracy compared with other algorithms are definitely proved.

Besides the above analysis, we count the number of successful runs and the average number of iterations in successful runs for the PS-FW over 12 benchmark functions, and the statistical results are presented in Table 11. In this section, a successful run means the algorithm can obtain the optimum within the 200,000 iterations. As shown in Table 11, the PS-FW can converge to the optimal solution in each of runs over the vast majority functions, which manifests the robustness of PS-FW in solving the optimization problems. In order to compare the convergence speed of PS-FW with other algorithms fairly, the average numbers of iterations in successful runs are compared over the six functions *f*_1_, *f*_4_, *f*_6_, *f*_7_, *f*_10_, and *f*_11_ introduced in Nickabadi et al.'s paper. According to the numerical results in Table 11, the PS-FW can converge to the optimal solution for all the six functions within 12,000 iterations, whereas the other algorithms have difficulty in obtaining the optimum for functions *f*_1_, *f*_6_, *f*_7_, and *f*_10_ after 200,000 iterations or can converge to the optimum for functions *f*_4_, *f*_11_ with a lot more iterations based on the convergence curves in the paper by Nickabadi et al. Therefore, we can argue that the robustness and convergence speed of PS-FW are superior to the other algorithms.

### 4.4. Experiments to Analyze the PS-FW Control Parameters

**Figure 6:**
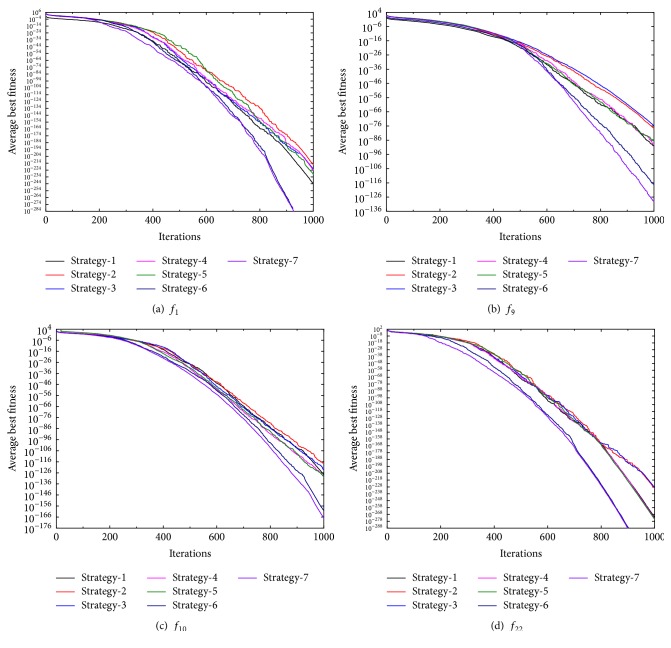
Convergence curves of PS-FW with different strategies for functions *f*_1_, *f*_9_, *f*_10_, and *f*_22_.

**Figure 7:**
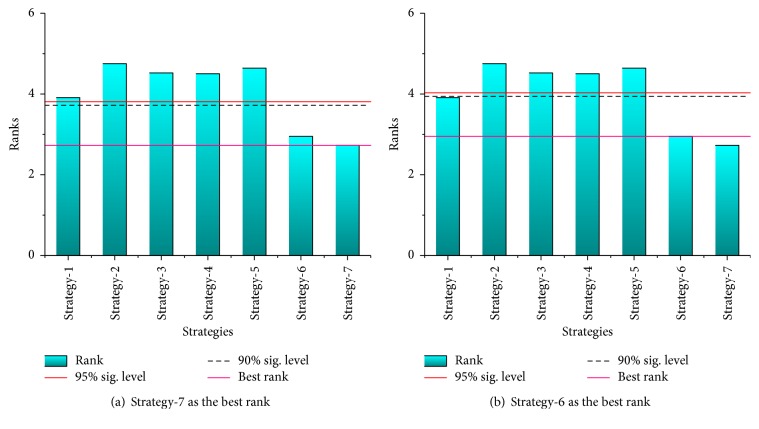
The bar chart of Bonferroni-Dunn test for different strategies over the mean of best solutions based on Table 15.

**Figure 8:**
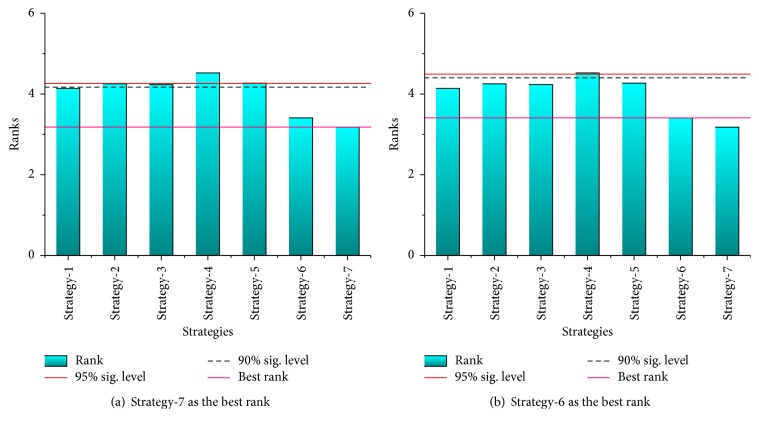
The bar chart of Bonferroni-Dunn test for different strategies over the standard deviation of best solutions based on Table 15.

In this section, we investigate the impact of the control parameters on the performance of PS-FW. From the previous introduction, the PS-FW has several control parameters including the parameters adopted from PSO and FWA. Here we only analyze the three main control parameters which are the control factors of explosion amplitudes *λ*_min_, *λ*_max_ and the number of mutation sparks num_*M*_. In order to test the impact of changes in control parameters on performance exhaustively, six different combinations of parameters were selected and experimented on. Each set of parameters corresponds to 20 runs based on 22 functions introduced in Table 1, and the dimensions of problems are set to 100. Moreover, the other parameters settings of PS-FW except *λ*_min_, *λ*_max_, and num_*M*_ are the same as those in Section 4.2. In addition, the six combinations of control parameters are represented as six optimization strategies, and their detailed parameters settings are shown in Table 12, and the control parameters of Section 4.2 are marked as Strategy-1 and are presented. As shown in Table 12, we take a contrasting method that changes a parameter and keeps the other parameters unchanged. Then the optimization results and the corresponding ranks of different strategies are shown in Tables 13 and 14, and the results focus on mean and standard deviation of best solutions obtained by different strategies. From the results of Tables 13 and 14, the PS-FW with Strategy-6 and Strategy-7 has the best performance for almost all the benchmark functions and can obtain the highest ranks over both the mean and standard deviation of best solutions. By adopting Strategy-6 and Strategy-7, the PS-FW can get the optimum of 16 functions for the whole 20 runs, especially including the functions *f*_1_, *f*_3_, *f*_6_, *f*_14_, *f*_19_, and *f*_22_ which cannot find the global best solutions by other optimization strategies of PS-FW. Therefore, the excellent performance of PS-FW with Strategy-6 and Strategy-7 proves the correctness of proposed mutation operator and indicates that increasing the number of mutation sparks can enhance the global search capability of the algorithm. However, according to the “no free lunch theorem” [42], there is no algorithm that can perform better than others on all the problems; hence the PS-FW with Strategy-6 and Strategy-7 has poor performance for function *f*_7_. It is because function *f*_7_ has a wide search scope so that the solutions have little changes in the later iterations if *λ*_min_ is small, which results in a relatively slow convergence speed for PS-FW despite the increase in the number of mutation sparks. For other strategies of PS-FW, the different strategies have their own advantages for various test functions, the PS-FW with Strategy-1 performs well for functions *f*_1_, *f*_3_, *f*_6_, *f*_9_, and *f*_19_, and the good solutions can be obtained by PS-FW over functions *f*_7_, *f*_16_ under Strategy-2 and Strategy-3. Meanwhile, the PS-FW with Strategy-4 and Strategy-5 works well in solving the functions *f*_10_ and *f*_22_. In addition, the PS-FW can obtain the optimum of functions *f*_2_, *f*_4_, *f*_5_, *f*_8_, *f*_12_, *f*_15_, *f*_17_, *f*_18_, *f*_20_, and *f*_21_ and keep outstanding performance in other functions under the whole seven strategies. Therefore, the robustness of the proposed algorithm is strongly proved. To compare the convergence speeds for different strategies of PS-FW, the convergence curves over several functions are shown in Figure 6. By observing the curves in Figure 6, the superiority of Strategy-6 and Strategy-7 in terms of convergence speed has been demonstrated, and the PS-FW with all strategies can converge to solutions that are very close to the optimums. Then we conduct the Friedman test and the Bonferroni-Dunn test for the mean and standard deviation of best solutions obtained by different optimization strategies, so as to determine the impact degree of each control parameter on the performance of PS-FW. The results of Friedman test for different strategies of PS-FW are shown in Table 15, and the results of Bonferroni-Dunn test in terms of mean and standard deviation based on Table 15 are presented in Figures 7 and 8.

According to the results of Friedman test in Table 15, the *p* value is lower than the level of significance considered *α* = 0.05 for both the mean and standard deviation of bets solutions, which indicates that the performance of seven strategies of PS-FW has the significant difference. By observing the ranks obtained by the Friedman test in Table 15, the PS-FW with Strategy-7 has the best performance followed by Strategy-6, Strategy-1, and so on, and the PS-FW with Strategy-2 performs the worst relative to other strategies over the average values of best solutions. In Bonferroni-Dunn test, the values of critical difference are the same as those in Section 4.2, and the lines of best rank and significant level are also drawn in Figures 7 and 8. Through checking the bars corresponding to the different strategies of PS-FW in Figure 7(a), the heights of bars for Strategy-1 to Strategy-5 exceed the lines of significant level. Hence Strategy-7 represents the best combination of control parameters among all the seven strategies, and the PS-FW with Strategy-7 performs significantly better than the other strategies except Strategy-6. In addition, the PS-FW with Strategy-6 has significant superiority compared with Strategy-2 to Strategy-5 over the average values of best solutions based on Figure 7(b). Besides, as shown in Figure 8, the hybrid algorithm with different strategies has relatively small gaps in standard deviation, Strategy-7 emerges as the best performer over the standard deviation of best solutions followed by Strategy-6, Strategy-1, and other strategies, and Strategy-4 has the worst performance.

Therefore, based on the analysis above, the solutions accuracy and convergence speed of PS-FW are determined by the control parameters *λ*_min_, *λ*_max_, and num_*M*_. Compared with *λ*_min_ and *λ*_max_, the number of mutation sparks has a greater impact on the performance of PS-FW. Hence we can appropriately increase the number of mutation sparks when solving the difficult multimodal global optimization problems. In addition, the value of *λ*_min_ can be increased properly for solving the optimization problems with large range such as function *f*_7_. Considering that the increase in the number of mutation sparks will make the computing time longer, to improve the computational efficiency, Strategy-1 which ranks third in seven strategies is used to conduct the experiments in Sections 4.2 and 4.3 in this paper. As expected, we should choose the suitable control parameters for various problems by taking all the aspects into consideration.

## 5. Conclusion

In this paper, a hybrid algorithm named PS-FW is proposed to solve the global optimization problems. In PS-FW, the exploitation capability is applied to find the optimal solution and make the hybrid algorithm converge quickly whereas the exploration ability of FWA is used to search for the better solutions in the entire feasible space. Moreover, the abandonment and supplement mechanism, the modified explosion operator, and the novel mutation operator are proposed to enhance both the global and local search ability of algorithm. Then the validity of PS-FW is confirmed by the 22 well-known high-dimensional benchmark functions. The results show that PS-FW is an efficacious, fast converging, and robust optimization algorithm by comparing with the PSO, FWA, stdPSO, CPSO, CLPSO, FIPS, Frankenstein, and ALWPSO over solving global optimization problems.

The future work is to refine the PS-FW by testing more complex high-dimensional optimization problems. Furthermore, we will try to apply the algorithm to multiobjective optimization problems and real-world problems such as spatial layout optimization, route optimization, and structural parameter optimization.

## Figures and Tables

**Algorithm 1:**
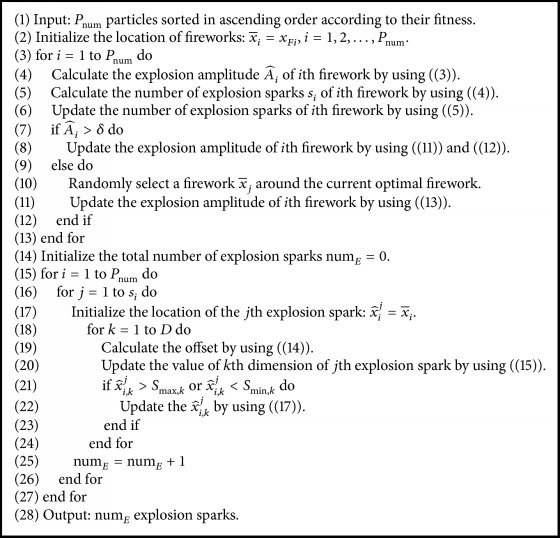
Generating explosion sparks by the explosion operator of PS-FW.

**Algorithm 2:**
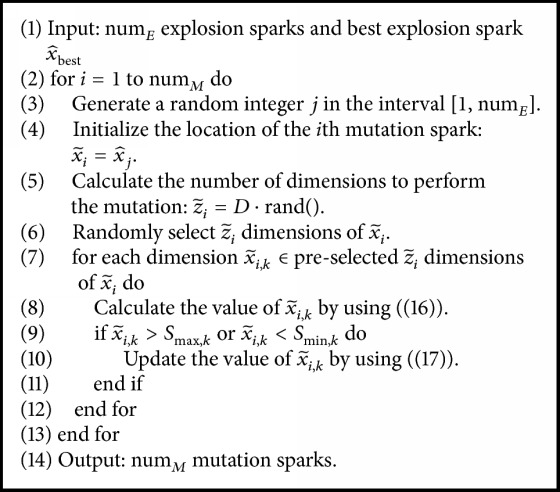
Generating mutation sparks by the mutation operator of PS-FW.

**Algorithm 3:**
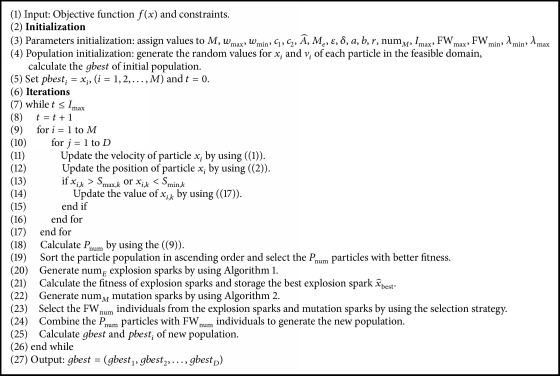
The main codes of PS-FW algorithm.

**Table 1:** The 22 high-dimensional benchmark functions.

Name	Function	Search space	Optimum
Sphere	$f_{1}\left(x\right)=\overset{D}{\underset{i=1}{\sum }}x_{i}^{2}$	[−100,100]^*D*^	0

Griewank	$f_{2}\left(x\right)=\frac{1}{4000}\overset{D}{\underset{i=1}{\sum }}x_{i}^{2}-\overset{D}{\underset{i=1}{\prod }}\cos\left(\frac{x_{i}}{\sqrt{i}}\right)+1$	[−600,600]^*D*^	0

Rosenbrock	$f_{3}\left(x\right)=\overset{D-1}{\underset{i=1}{\sum }}\left[100{\left(x_{i+1}-x_{i}^{2}\right)}^{2}+{\left(x_{i}-1\right)}^{2}\right]$	[−5,10]^*D*^	0

Rastrigin	$f_{4}\left(x\right)=10D+\overset{D}{\underset{i=1}{\sum }}\left[x_{i}^{2}-10\cos\left(2\pi x_{i}\right)\right]$	[−5.12,5.12]^*D*^	0

Noncontinuous Rastrigin	$f_{5}(x)=\overset{D}{\underset{i=1}{\sum }}y_{i}^{2}-10\cos\left(2\pi y_{i}\right)+10$ $y_{i}=\left\{\begin{array}{cc} x_{i} & \left|x_{i}\right|<0.5\\ \frac{round\left(2x_{i}\right)}{2} & \left|x_{i}\right|\geq 0.5 \end{array}\right.$	[−5,10]^*D*^	0

Ackley	$f_{6}\left(x\right)=-20\exp\left(-0.2\sqrt{\frac{1}{D}\overset{D}{\underset{i=1}{\sum }}x_{i}^{2}}\right)-\exp\left(\frac{1}{D}\overset{D}{\underset{i=1}{\sum }}\cos\left(2\pi x_{i}\right)\right)+20+e$	[−30,30]^*D*^	0

Rotated Hyper-Ellipsoid	$f_{7}\left(x\right)=\overset{D}{\underset{i=1}{\sum }}\;\overset{i}{\underset{j=1}{\sum }}x_{j}^{2}$	[−65536,65536]^*D*^	0

Noisy Quadric	$f_{8}\left(x\right)=\overset{D}{\underset{i=1}{\sum }}ix^{4}+rand$	[−1.28,1.28]^*D*^	0

Schwefel's problem 2.21	$f_{9}\left(x\right)=\underset{1\leq i\leq D}{\max}\left|x_{i}\right|$	[−100,100]^*D*^	0

Schwefel's problem 2.22	$f_{10}\left(x\right)=\overset{D}{\underset{i=1}{\sum }}\left|x_{i}\right|+\overset{D}{\underset{i=1}{\prod }}\left|x_{i}\right|$	[−100,100]^*D*^	0

Schwefel's problem 2.26	$f_{11}\left(x\right)=\overset{D}{\underset{i=1}{\sum }}-x_{i}\sin\left(\sqrt{\left|x_{i}\right|}\right)$	[−500,500]^*d*^	−418.9829*D*

Step	$f_{12}\left(x\right)=\overset{D}{\underset{i=1}{\sum }}{\left(\left[x_{i}+0.5\right]\right)}^{2}$	[−10,10]^*D*^	0

Levy	$f_{13}\left(x\right)={sin}^{2}\left(\pi y_{1}\right)+\overset{D-1}{\underset{i=1}{\sum }}{\left(y_{i}-1\right)}^{2}\left[1+10\;{sin}^{2}\left(\pi y_{i}+1\right)\right]$ +(*y*_*D*_ − 1)^2^[1 + sin^2^(2*πy*_*D*_)] $y_{i}=1+\frac{x_{i}-1}{4}$	[−10,10]^*D*^	0

Powell Sum	$f_{14}\left(x\right)=\overset{D}{\underset{i=1}{\sum }}{\left|x_{i}\right|}^{i+1}$	[−1,1]^*D*^	0

Sum squares	$f_{15}\left(x\right)=\overset{D}{\underset{i=1}{\sum }}ix_{i}^{2}$	[−10,10]^*D*^	0

Zakharov	$f_{16}\left(x\right)=\overset{D}{\underset{i=1}{\sum }}x_{i}^{2}+{\left(\sum_{i=1}^{D}0.5ix_{i}\right)}^{2}+{\left(\sum_{i=1}^{D}0.5ix_{i}\right)}^{4}$	[−5,10]^*D*^	0

Mishra 7	$f_{17}\left(x\right)={\left(\prod_{i=1}^{D}x_{i}-D!\right)}^{2}$	[−*D*, *D*]^*D*^	0

Weierstrass	$f_{18}\left(x\right)=\overset{D}{\underset{i=1}{\sum }}\left[\overset{k_{max}}{\underset{k=0}{\sum }}\left(a^{k}\cos\left(2\pi b^{k}\left(x_{i}+0.5\right)\right)\right)-D\overset{k_{max}}{\underset{k=0}{\sum }}a^{k}\cos\left(\pi b^{k}\right)\right]$	[−0.5,0.5]^*D*^	0
	*a* = 0.5, *b* = 3, *k*_max_ = 20		

Bent-Cigar	$f_{19}\left(x\right)=x_{1}^{2}+10^{6}\overset{D}{\underset{i=1}{\sum }}x_{i}^{2}$	[−100,100]^*D*^	0

Trigonometric 2	$f_{20}\left(x\right)=1+\overset{D}{\underset{i=1}{\sum }}8\;{sin}^{2}\left[7{\left(x_{i}-0.9\right)}^{2}\right]+6\;{sin}^{2}\left[14{\left(x_{i}-0.9\right)}^{2}\right]+{\left(x-0.9\right)}^{2}$	[−500,500]^*D*^	1

Quintic	$f_{21}\left(x\right)=\overset{D}{\underset{i=1}{\sum }}\left|x_{i}^{5}-3x_{i}^{4}+4x_{i}^{3}+2x_{i}^{2}-10x_{i}-4\right|$	[−10,10]^*D*^	0

Mishra 11	$f_{22}\left(x\right)={\left[\frac{1}{D}\sum_{i=1}^{D}\left|x_{i}\right|+{\left(\prod_{i=1}^{D}\left|x_{i}\right|\right)}^{1/D}\right]}^{2}$	[−10,10]^*D*^	0

**Table 2:** The parameter setting of the algorithms.

Algorithm	Parameter settings
PSO	$w\left(t\right)=w_{max}-t\frac{w_{max}-w_{min}}{I_{max}}$, *w*_max_ = 0.95,
	*w*_min_ = 0.4, *c*_1_ = *c*_2_ = 1.45

FWA	$\hat{A}=40$, *Me* = 50, *a* = 0.04, *b* = 0.8,
	num_*M*_ = 30, *ε* = 1*E* − 100

PS-FW	$w(t)=w_{max}-t\frac{w_{max}-w_{min}}{I_{max}}$, *w*_max_ = 0.95,
	*w*_min_ = 0.4, *c*_1_ = *c*_2_ = 1.45, $\hat{A}=40$,
	*Me* = 50, *a* = 0.04, *b* = 0.8, num_*M*_ = 30,
	*ε* = 1*E* − 100, *δ* = 1*E* − 6, *λ*_min_ = 1*E* − 25,
	*λ*_max_ = 1, FW_max_ = 30, FW_min_ = 20, *r* = 2

**Table 3:** Comparison of the optimization results obtained by PS-FW, PSO, and FWA with *D* = 30 for functions *f*_1_ to *f*_22_ (the best ranks are marked in bold).

*f*	*D*		PSO	FWA	PS-FW
*f*_1_	30	Mean	8.8371*E* + 01	1.3360*E* − 151	5.8928*E* − 264
		Std	4.3475*E* + 01	5.8057*E* − 151	0
		Rank	3	2	1

*f*_2_	30	Mean	7.1542*E* − 02	0	0
		Std	1.2385*E* − 01	0	0
		Rank	2	1	1

*f*_3_	30	Mean	5.5766*E* + 02	2.6882*E* + 01	0
		Std	7.4828*E* + 02	8.3997*E* − 01	0
		Rank	3	2	1

*f*_4_	30	Mean	6.6547*E* + 01	0	0
		Std	3.6430*E* + 01	0	0
		Rank	2	1	1

*f*_5_	30	Mean	6.5810*E* + 01	0	0
		Std	4.0117*E* + 01	0	0
		Rank	2	1	1

*f*_6_	30	Mean	0	0	0
		Std	0	0	0
		Rank	1	1	1

*f*_7_	30	Mean	1.4156*E* + 04	7.6585*E* − 83	4.5128*E* − 122
		Std	1.0006*E* + 04	3.3383*E* − 82	1.8821*E* − 121
		Rank	3	2	1

*f*_8_	30	Mean	1.0419*E* − 03	9.6596*E* − 304	0
		Std	1.0584*E* − 03	0	0
		Rank	3	2	1

*f*_9_	30	Mean	6.3165*E* − 01	7.4698*E* − 54	3.1588*E* − 97
		Std	6.0679*E* − 01	2.3638*E* − 53	1.2719*E* − 96
		Rank	3	2	1

*f*_10_	30	Mean	1.5661*E* + 01	3.2521*E* − 78	1.8666*E* − 137
		Std	5.0924*E* + 00	1.1460*E* − 77	8.0013*E* − 137
		Rank	3	2	1

*f*_11_	30	Mean	−7.2662*E* + 03	−1.0511*E* + 04	−1.2483*E* + 04
		Std	6.7867*E* + 02	1.9893*E* + 02	1.2661*E* + 02
		Rank	3	2	1

*f*_12_	30	Mean	6.9734*E* − 01	6.6542*E* − 01	0
		Std	2.8586*E* − 01	5.0080*E* − 01	0
		Rank	3	2	1

*f*_13_	30	Mean	1.7831*E* + 01	6.5460*E* + 00	1.4998*E* − 32
		Std	8.6204*E* + 00	8.6700*E* − 01	0
		Rank	3	2	1

*f*_14_	30	Mean	6.6576*E* − 08	4.5613*E* − 191	2.1563*E* − 291
		Std	5.4575*E* − 08	0	0
		Rank	3	2	1

*f*_15_	30	Mean	0	0	0
		Std	0	0	0
		Rank	1	1	1

*f*_16_	30	Mean	2.8937*E* + 02	1.5997*E* − 45	1.5471*E* − 111
		Std	1.5937*E* + 02	3.5711*E* − 45	6.0668*E* − 111
		Rank	3	2	1

*f*_17_	30	Mean	0	9.8737*E* + 44	0
		Std	0	4.3038*E* + 45	0
		Rank	1	2	1

*f*_18_	30	Mean	1.5069*E* + 01	0	0
		Std	4.0495*E* + 00	0	0
		Rank	2	1	1

*f*_19_	30	Mean	2.8450*E* + 07	1.0123*E* − 145	1.8302*E* − 252
		Std	1.2385*E* + 08	3.1288*E* − 145	0
		Rank	3	2	1

*f*_20_	30	Mean	3.8005*E* + 02	4.2079*E* + 01	1
		Std	8.5739*E* + 01	4.6125*E* + 00	0
		Rank	3	2	1

*f*_21_	30	Mean	4.5577*E* + 01	1.71130*E* + 01	0
		Std	2.3091*E* + 01	2.1499*E* + 00	0
		Rank	3	2	1

*f*_22_	30	Mean	7.0166*E* − 01	1.1989*E* − 149	3.5102*E* − 292
		Std	5.9846*E* − 01	5.2258*E* − 149	0
		Rank	3	2	1

Average rank			2.5455	1.7273	1
Overall rank			3	2	1

**Table 4:** Comparison of the optimization results obtained by PS-FW, PSO, and FWA with *D* = 60 for functions *f*_1_ to *f*_22_ (the best ranks are marked in bold).

*f*	*D*		PSO	FWA	PS-FW
*f*_1_	60	Mean	4.1677*E* + 03	2.1235*E* − 146	2.4481*E* − 248
		Std	4.4284*E* + 03	6.3705*E* − 146	0
		Rank	3	2	1

*f*_2_	60	Mean	3.2482*E* + 00	0	0
		Std	9.6094*E* − 01	0	0
		Rank	2	1	1

*f*_3_	60	Mean	7.1638*E* + 04	4.5073*E* + 01	9.2568*E* − 30
		Std	5.5811*E* + 04	1.8390*E* + 01	1.9330*E* − 29
		Rank	3	2	1

*f*_4_	60	Mean	3.2219*E* + 02	0	0
		Std	4.1863*E* + 01	0	0
		Rank	2	1	1

*f*_5_	60	Mean	3.7498*E* + 02	0	0
		Std	5.3191*E* + 01	0	0
		Rank	2	1	1

*f*_6_	60	Mean	1.3162*E* + 01	0	7.1054*E* − 16
		Std	1.1773*E* + 00	0	1.4211*E* − 15
		Rank	3	1	2

*f*_7_	60	Mean	3.2017*E* + 04	4.9633*E* − 68	1.2294*E* − 93
		Std	1.4529*E* + 04	1.48899*E* − 67	4.9341*E* − 93
		Rank	3	2	1

*f*_8_	60	Mean	1.1343*E* + 00	1.2096*E* − 288	0
		Std	3.2234*E* + 00	0	0
		Rank	3	2	1

*f*_9_	60	Mean	2.6902*E* + 01	4.4049*E* − 51	1.5914*E* − 92
		Std	5.4555*E* + 00	1.3214*E* − 50	4.8189*E* − 92
		Rank	3	2	1

*f*_10_	60	Mean	5.5140*E* + 01	1.35612*E* − 73	3.9617*E* − 130
		Std	2.1038*E* + 01	4.06287*E* − 73	1.7268*E* − 129
		Rank	3	2	1

*f*_11_	60	Mean	−1.1892*E* + 04	−1.8005*E* + 04	−2.4998*E* + 04
		Std	1.1022*E* + 03	1.4727*E* + 03	1.7201*E* + 02
		Rank	3	2	1

*f*_12_	60	Mean	3.4856*E* + 01	1.9695*E* + 00	0
		Std	5.9316*E* + 01	7.7525*E* − 01	0
		Rank	3	2	1

*f*_13_	60	Mean	6.2329*E* + 01	1.5355*E* + 01	1.4998*E* − 32
		Std	2.0956*E* + 01	5.4415*E* + 00	0
		Rank	3	2	1

*f*_14_	60	Mean	2.2365*E* − 07	1.6432*E* − 187	1.5707*E* − 278
		Std	2.3968*E* − 07	0	0
		Rank	3	2	1

*f*_15_	60	Mean	0	0	0
		Std	0	0	0
		Rank	1	1	1

*f*_16_	60	Mean	8.0994*E* + 02	1.7189*E* − 38	6.8924*E* − 104
		Std	3.0726*E* + 02	5.15482*E* − 38	2.9641*E* − 103
		Rank	3	2	1

*f*_17_	60	Mean	0	2.4945*E* + 145	0
		Std	0	5.7208*E* + 145	0
		Rank	1	2	1

*f*_18_	60	Mean	3.9564*E* + 01	0	0
		Std	5.3138*E* + 00	0	0
		Rank	2	1	1

*f*_19_	60	Mean	5.7753*E* + 08	6.6011*E* − 137	4.5120*E* − 251
		Std	2.7159*E* + 08	1.9631*E* − 136	0
		Rank	3	2	1

*f*_20_	60	Mean	5.3645*E* + 03	1.4665*E* + 02	1
		Std	6.2256*E* + 03	2.8947*E* + 01	0
		Rank	3	2	1

*f*_21_	60	Mean	1.9709*E* + 02	4.8085*E* + 01	0
		Std	2.8605*E* + 01	7.7355*E* + 00	0
		Rank	3	2	1

*f*_22_	60	Mean	1.5314*E* + 00	1.5711*E* − 142	1.3216*E* − 280
		Std	5.9245*E* − 01	4.7133*E* − 142	0
		Rank	3	2	1

Average rank			2.6364	1.7273	1.0455
Overall rank			3	2	1

**Table 5:** Comparison of the optimization results obtained by PS-FW, PSO, and FWA with *D* = 100 for functions *f*_1_ to *f*_22_ (the best ranks are marked in bold).

*f*	*D*		PSO	FWA	PS-FW
*f*_1_	100	Mean	6.3501*E* + 03	1.7672*E* − 142	9.7833*E* − 245
		Std	2.9204*E* + 03	4.3844*E* − 142	0
		Rank	3	2	1

*f*_2_	100	Mean	1.1830*E* + 02	0	0
		Std	5.1822*E* + 01	0	0
		Rank	2	1	1

*f*_3_	100	Mean	1.7018*E* + 05	8.3094*E* + 01	1.0341*E* − 26
		Std	6.6940*E* + 04	2.2198*E* + 01	3.8500*E* − 26
		Rank	3	2	1

*f*_4_	100	Mean	4.7288*E* + 02	0	0
		Std	1.0713*E* + 02	0	0
		Rank	2	1	1

*f*_5_	100	Mean	5.1626*E* + 02	0	0
		Std	1.4819*E* + 02	0	0
		Rank	2	1	1

*f*_6_	100	Mean	1.3582*E* + 01	0	1.0659*E* − 15
		Std	2.3679*E* + 00	0	1.6281*E* − 15
		Rank	3	1	2

*f*_7_	100	Mean	2.7218*E* + 06	2.70634*E* − 58	2.1860*E* − 71
		Std	8.2328*E* + 05	8.11903*E* − 58	4.7535*E* − 71
		Rank	3	2	1

*f*_8_	100	Mean	1.4283*E* + 01	1.5868*E* − 280	0
		Std	3.8266*E* + 01	0	0
		Rank	3	2	1

*f*_9_	100	Mean	2.7189*E* + 01	4.2938*E* − 46	1.1555*E* − 90
		Std	5.0564*E* + 00	1.1238*E* − 45	2.7315*E* − 90
		Rank	3	2	1

*f*_10_	100	Mean	1.2486*E* + 02	2.64613*E* − 69	2.2792*E* − 128
		Std	2.3963*E* + 01	7.93838*E* − 69	9.7764*E* − 128
		Rank	3	2	1

*f*_11_	100	Mean	−1.5770*E* + 04	−2.4526*E* + 04	−4.1743*E* + 04
		Std	1.2531*E* + 03	1.6861*E* + 03	4.3502*E* + 02
		Rank	3	2	1

*f*_12_	100	Mean	1.2670*E* + 02	4.2335*E* + 00	0
		Std	4.8966*E* + 01	1.40825853	0
		Rank	3	2	1

*f*_13_	100	Mean	2.4848*E* + 02	3.1912*E* + 01	1.4998*E* − 32
		Std	6.1955*E* + 01	7.6762*E* + 00	0
		Rank	3	2	1

*f*_14_	100	Mean	4.7875*E* − 07	6.5204*E* − 175	6.4751*E* − 275
		Std	6.7428*E* − 07	0	0
		Rank	3	2	1

*f*_15_	100	Mean	0	0	0
		Std	0	0	0
		Rank	1	1	1

*f*_16_	100	Mean	1.4995*E* + 03	1.9628*E* − 14	2.4731*E* − 93
		Std	5.8180*E* + 02	5.86607*E* − 14	8.4009*E* − 93
		Rank	3	2	1

*f*_17_	100	Mean	0	2.0047*E* + 232	0
		Std	0	6.7205*E* + 232	0
		Rank	1	2	1

*f*_18_	100	Mean	6.8687*E* + 01	0	0
		Std	1.3221*E* + 01	0	0
		Rank	2	1	1

*f*_19_	100	Mean	1.4528*E* + 10	3.3916*E* − 130	9.0096*E* − 250
		Std	1.2994*E* + 10	9.8384*E* − 130	0
		Rank	3	2	1

*f*_20_	100	Mean	9.0245*E* + 03	2.6557*E* + 02	1
		Std	3.8036*E* + 03	4.7674*E* + 01	0
		Rank	3	2	1

*f*_21_	100	Mean	4.0256*E* + 03	9.1975*E* + 01	0
		Std	1.6131*E* + 04	1.7966*E* + 01	0
		Rank	3	2	1

*f*_22_	100	Mean	1.6273*E* + 00	4.0925*E* − 137	4.9253*E* − 273
		Std	4.1513*E* − 01	3.2175*E* − 137	0
		Rank	3	2	1

Average rank			2.6364	1.7273	1.0455
Overall rank			3	2	1

**Table 6:** The benchmark functions with shift optima.

Name	Original optima	Shift optima
Sphere	[0,0,…, 0]	[70,70,…, 70]
Griewank	[0,0,…, 0]	[70,70,…, 70]
Rastrigin	[0,0,…, 0]	[3,3,…, 3]
Noncontinuous Rastrigin	[0,0,…, 0]	[5,5,…, 5]
Ackley	[0,0,…, 0]	[20,20,…, 20]
Rotated Hyper-Ellipsoid	[0,0,…, 0]	[70,70,…, 70]
Schwefel's problem 2.21	[0,0,…, 0]	[70,70,…, 70]
Schwefel's problem 2.22	[0,0,…, 0]	[70,70,…, 70]
Step	[−0.5, −0.5,…, −0.5]	[5,5,…, 5]
Levy	[1,1,…, 1]	[5,5,…, 5]
Sum squares	[0,0,…, 0]	[5,5,…, 5]
Zakharov	[0,0,…, 0]	[5,5,…, 5]
Bent-Cigar	[0,0,…, 0]	[70,70,…, 70]
Trigonometric 2	[0.9,0.9,…, 0.9]	[70,70,…, 70]
Mishra 11	[0,0,…, 0]	[5,5,…, 5]

**Table 7:** Comparison of the optimization results obtained by PS-FW, PSO, and FWA for functions in Table 6 (the best ranks are marked in bold).

*f*	*D*		PSO	FWA	PS-FW
*f*_1_	30	Mean	1.0851*E* + 03	2.2555*E* + 00	0
		Std	1.1893*E* + 03	3.8190*E* − 01	0
		Rank	3	2	1

*f*_2_	30	Mean	4.7829*E* + 00	6.2867*E* − 01	0
		Std	1.5089*E* + 00	5.3523*E* − 02	0
		Rank	3	2	1

*f*_4_	30	Mean	1.2559*E* + 02	9.8052*E* + 00	0
		Std	4.7596*E* + 01	1.6323*E* + 00	0
		Rank	3	2	1

*f*_5_	30	Mean	1.6140*E* + 02	2.2289*E* + 01	0
		Std	3.7649*E* + 01	2.7981*E* + 00	0
		Rank	3	2	1

*f*_6_	30	Mean	1.0739*E* + 03	7.0977*E* + 00	0
		Std	1.1986*E* + 03	4.3511*E* − 01	0
		Rank	3	2	1

*f*_7_	30	Mean	1.5716*E* + 04	2.2295*E* + 03	4.45263*E* − 65
		Std	8.7224*E* + 03	2.4129*E* + 02	2.87935*E* − 65
		Rank	3	2	1

*f*_9_	30	Mean	4.7379*E* + 01	2.1052*E* + 01	8.96847*E* − 72
		Std	1.5948*E* + 01	1.4289*E* + 00	1.31198*E* − 71
		Rank	3	2	1

*f*_10_	30	Mean	1.6846*E* + 03	2.2370*E* + 02	0
		Std	2.6627*E* + 02	7.4690*E* + 01	0
		Rank	3	2	1

*f*_12_	30	Mean	1.1359*E* + 02	2.1375*E* + 01	0
		Std	4.1907*E* + 01	2.9107*E* + 00	0
		Rank	3	2	1

*f*_13_	30	Mean	3.2776*E* + 02	6.4154*E* + 01	1.4998*E* − 32
		Std	8.5157*E* + 01	1.0092*E* + 01	0
		Rank	3	2	1

*f*_15_	30	Mean	0	2.9887*E* − 04	0
		Std	0	1.3027*E* − 03	0
		Rank	1	2	1

*f*_16_	30	Mean	8.0214*E* + 00	3.1159*E* + 02	1.53313*E* − 06
		Std	8.1866*E* + 00	2.0373*E* + 02	1.06687*E* − 06
		Rank	2	3	1

*f*_19_	30	Mean	2.4875*E* + 09	2.2700*E* + 08	0
		Std	1.3163*E* + 09	2.7319*E* + 07	0
		Rank	3	2	1

*f*_20_	30	Mean	2.0564*E* + 03	9.2562*E* + 02	1
		Std	7.9311*E* + 02	7.6748*E* + 01	0
		Rank	3	2	1

*f*_22_	30	Mean	1.7217*E* + 00	1.4009*E* + 00	0
		Std	1.1645*E* + 00	4.6093*E* − 01	0
		Rank	3	2	1

Average rank			2.8000	2.0667	1
Overall rank			3	2	1

**Table 8:** Comparison of successful rates and average number of iterations for PS-FW, PSO, and FWA with *τ* = 10^−4^ for function *f*_15_ and *τ* = 10^1^ for other functions (the best ranks are marked in bold).

*f*	PSO	FWA	PS-FW
*f*_1_			
ST	0	20	20
Rank	2	1	1
AI	*U*	201.7	28.4
Rank	3	2	1
*f*_2_			
ST	19	20	20
Rank	2	1	1
AI	9.6	4.6	2.8
Rank	3	2	1
*f*_4_			
ST	0	11	20
Rank	3	2	1
AI	*U*	584.8	228.8
Rank	3	2	1
*f*_5_			
ST	0	0	20
Rank	2	2	1
AI	*U*	*U*	104.9
Rank	2	2	1
*f*_6_			
ST	0	20	20
Rank	2	1	1
AI	*U*	343	9.8
Rank	3	2	1
*f*_7_			
ST	0	0	20
Rank	2	2	1
AI	*U*	*U*	93.8
Rank	2	2	1
*f*_9_			
ST	0	0	20
Rank	2	2	1
AI	*U*	*U*	26.7
Rank	2	2	1
*f*_10_			
ST	0	0	20
Rank	2	2	1
AI	*U*	*U*	41.1
Rank	2	2	1
*f*_12_			
ST	0	0	20
Rank	2	2	1
AI	*U*	*U*	11.8
Rank	2	2	1
*f*_13_			
ST	0	0	20
Rank	2	2	1
AI	*U*	*U*	3.5
Rank	2	2	1
*f*_15_			
ST	20	19	20
Rank	1	2	1
AI	505.3	679.6	13.1
Rank	2	3	1
*f*_16_			
ST	16	0	20
Rank	2	3	1
AI	224	*U*	208.7
Rank	2	3	1
*f*_19_			
ST	0	0	20
Rank	2	2	1
AI	*U*	*U*	208.9
Rank	2	2	1
*f*_20_			
ST	0	0	20
Rank	2	2	1
AI	*U*	*U*	160.8
Rank	2	2	1
*f*_22_			
ST	20	20	20
Rank	1	1	1
AI	94.2	123.2	9.3
Rank	2	3	1

Average rank of ST	1.9	1.8	1
Overall rank of AI	2.3	2.2	1

**Table 9:** Comparison of the optimization results obtained by PS-FW and six PSO variants (the best ranks are marked in bold).

*f*(*x*)	PS-FW	stdPSO	CPSO	CLPSO	FIPS	Frankenstein	AIWPSO
*f*_1_							
Mean	0	5.198*E* − 40	5.146*E* − 13	4.894*E* − 39	4.588*E* − 27	2.409*E* − 16	3.370*E* − 134
Rank	1	3	7	4	5	6	2
Std	0	1.1301*E* − 78	7.7588*E* − 25	6.7814*E* − 78	1.9577*E* − 53	2.0047*E* − 31	5.1722*E* − 267
Rank	1	3	7	4	5	6	2
*f*_2_							
Mean	0	2.1625*E* − 02	2.1245*E* − 02	0	2.4776*E* − 04	1.4736*E* − 03	2.8524*E* − 02
Rank	1	5	4	1	2	3	6
Std	0	4.5019*E* − 04	6.3144*E* − 04	0	1.8266*E* − 06	1.2846*E* − 05	7.6640*E* − 04
Rank	1	4	5	1	2	3	6
*f*_3_							
Mean	0	2.5404*E* + 01	8.2648*E* − 01	1.3217*E* + 01	2.6714*E* + 01	2.8156*E* + 01	2.5003*E* + 00
Rank	1	5	2	4	6	7	3
Std	0	5.9031*E* + 02	2.3449*E* + 00	2.1480*E* + 02	2.0025*E* + 02	2.3132*E* + 02	1.5978*E* + 01
Rank	1	7	2	5	4	6	3
*f*_4_							
Mean	0	3.4757*E* + 01	3.6007*E* − 13	0	5.8502*E* + 01	7.3836*E* + 01	1.6583*E* − 01
Rank	1	4	2	1	5	6	3
Std	0	1.0636*E* + 02	1.5035*E* − 24	0	1.9185*E* + 02	3.7055*E* + 02	2.1051*E* − 01
Rank	1	4	2	1	5	6	3
*f*_5_							
Mean	0	2.0956*E* + 01	5.3717*E* − 13	1.3333*E* − 01	6.1883*E* + 01	7.0347*E* + 01	1.1842*E* − 16
Rank	1	5	3	4	6	7	2
Std	0	1.8327*E* + 02	5.9437*E* − 24	1.1954*E* − 01	1.4013*E* + 02	2.9600*E* + 02	4.2073*E* − 31
Rank	1	6	3	4	5	7	2
*f*_6_							
Mean	0	1.4921*E* − 14	1.6091*E* − 07	9.2371*E* − 15	1.3856*E* − 14	2.1792*E* − 09	6.9870*E* − 15
Rank	1	5	7	3	4	6	2
Std	0	1.8628*E* − 29	7.8608*E* − 14	6.6156*E* − 30	2.3227*E* − 29	1.7187*E* − 18	4.2073*E* − 31
Rank	1	4	7	3	5	6	2
*f*_7_							
Mean	0	1.4582*E* + 00	1.8889*E* + 03	1.9217*E* + 02	9.4634*E* + 00	1.7315*E* + 02	1.9570*E* − 10
Rank	1	3	7	6	4	5	2
Std	0	1.1783*E* + 00	9.9106*E* + 06	3.8433*E* + 03	2.5976*E* + 01	9.1577*E* + 03	1.2012*E* − 19
Rank	1	3	7	5	4	6	2
*f*_8_							
Mean	0	1.2375*E* − 02	1.0764*E* − 02	4.0642*E* − 03	3.3047*E* − 03	4.1690*E* − 03	5.5241*E* − 03
Rank	1	7	6	3	2	4	5
Std	0	2.3107*E* − 05	2.7698*E* − 05	9.6184*E* − 07	8.6680*E* − 07	2.4012*E* − 06	1.5358*E* − 05
Rank	1	6	7	3	2	4	5
*f*_10_							
Mean	0	3.4621*E* − 26	5.4282*E* − 14	9.9748*E* − 39	2.6033*E* + 02	5.1953*E* + 04	1.8317*E* − 137
Rank	1	4	5	3	6	7	2
Std	0	4.0873*E* − 51	8.2868*E* − 27	3.7661*E* − 84	2.1785*E* + 04	1.1136*E* + 09	3.4534*E* − 273
Rank	1	4	5	3	6	7	2
*f*_11_							
Mean	−1.2542*E* + 04	−1.0995*E* + 04	−1.2127*E* + 04	−1.2546*E* + 04	−1.1052*E* + 04	−1.1221*E* + 04	−1.2569*E* + 04
Rank	3	7	5	2	6	4	1
Std	1.4900*E* + 02	1.3753*E* + 05	3.3795*E* + 04	4.2567*E* + 03	9.4421*E* + 05	2.7708*E* + 05	1.1409*E* − 25
Rank	2	5	4	3	7	6	1
*f*_12_							
Mean	0	0	0	0	0	0	0
Rank	1	1	1	1	1	1	1
Std	0	0	0	0	0	0	0
Rank	1	1	1	1	1	1	1
*f*_13_							
Mean	1.4998*E* − 32	1.1422*E* − 29	2.0913*E* − 15	1.4998*E* − 32	1.0273*E* − 28	5.5136*E* − 18	1.4998*E* − 32
Rank	1	2	5	1	3	4	1
Std	0	3.2335*E* − 57	1.2954*E* − 29	1.2398*E* − 94	1.0052*E* − 56	1.4501*E* − 34	1.2398*E* − 94
Rank	1	3	6	2	4	5	2

**Table 10:** The results of Friedman test for the PS-FW and other PSO variants over the mean and standard deviation of best solutions based on Table 9 (the best ranks are marked in bold).

	Mean	Std
Results		
*N*	12	12
Chi-square	35.33	37.18
*p* value	3.72*E* − 06	1.62*E* − 06
Friedman ranks of Algorithms		
PS-FW	1.58	1.5
stdPso	4.83	4.67
CPSO	5.08	5.17
CLPSO	3.17	3.25
FIPS	4.75	4.67
Frankenstein	5.58	5.75
AIWPSO	3	3

**Table 11:** The statistical results of PS-FW in terms of success rate and average number of iterations in successful runs for 12 benchmark functions.

Functions	ST	AT
*f*_1_	30	3828.0
*f*_2_	30	882.6
*f*_3_	30	11266.5
*f*_4_	30	1853.8
*f*_5_	30	2134.7
*f*_6_	30	755.1
*f*_7_	30	5910.4
*f*_8_	30	2281.1
*f*_10_	30	6304.7
*f*_11_	29	1100.5
*f*_12_	30	7516.0
*f*_13_	0	*U*

**Table 12:** The detailed parameters settings of the different optimization strategies for PS-FW (the square brackets represent the rounding operations).

Strategies	*λ*_max_	*λ*_min_	num_*M*_
Strategy-1	1	1*E* − 25	30
Strategy-2	1	1*E* − 10	30
Strategy-3	1	0.1	30
Strategy-4	0.8	1*E* − 25	30
Strategy-5	0.6	1*E* − 25	30
Strategy-6	1	1*E* − 25	[0.5 · num_*E*_]
Strategy-7	1	1*E* − 25	[0.7 · num_*E*_]

**Table 13:** The mean, standard deviation, and corresponding ranks of best solutions obtained by different optimization strategies of PS-FW for functions *f*_1_ to *f*_13_ (the best ranks are marked in bold).

*f*(*x*)	Strategy-1	Strategy-2	Strategy-3	Strategy-4	Strategy-5	Strategy-6	Strategy-7
*f*_1_							
Mean	9.7833*E* − 245	6.6617*E* − 217	8.1065*E* − 224	1.4930*E* − 224	6.8133*E* − 231	0	0
Rank	2	6	5	4	3	1	1
Std	0	0	0	0	0	0	0
Rank	1	1	1	1	1	1	1
*f*_2_							
Mean	0	0	0	0	0	0	0
Rank	1	1	1	1	1	1	1
Std	0	0	0	0	0	0	0
Rank	1	1	1	1	1	1	1
*f*_3_							
Mean	1.0341*E* − 26	7.1483*E* − 16	2.5737*E* − 13	1.3156*E* − 09	2.2836*E* − 09	0	0
Rank	2	3	4	5	6	1	1
Std	3.8500*E* − 26	1.3157*E* − 15	7.1641*E* − 13	4.2629*E* − 09	4.5987*E* − 09	0	0
Rank	2	3	4	5	6	1	1
*f*_4_							
Mean	0	0	0	0	0	0	0
Rank	1	1	1	1	1	1	1
Std	0	0	0	0	0	0	0
Rank	1	1	1	1	1	1	1
*f*_5_							
Mean	0	0	0	0	0	0	0
Rank	1	1	1	1	1	1	1
Std	0	0	0	0	0	0	0
Rank	1	1	1	1	1	1	1
*f*_6_							
Mean	7.1054*E* − 16	2.3093*E* − 15	1.4211*E* − 15	2.3093*E* − 15	2.4869*E* − 15	0	0
Rank	2	4	3	4	5	1	1
Std	1.4211*E* − 15	1.6945*E* − 15	1.7405*E* − 15	1.6945*E* − 15	1.6281*E* − 15	0	0
Rank	2	4	5	4	3	1	1
*f*_7_							
Mean	2.1860*E* − 71	7.0151*E* − 123	3.5034*E* − 126	2.7732*E* − 62	2.0900*E* − 65	5.7053*E* − 83	2.3724*E* − 87
Rank	5	2	1	7	6	4	3
Std	4.7535*E* − 71	1.8052*E* − 122	1.2502*E* − 125	1.2084*E* − 61	9.0599*E* − 65	5.7716*E* − 83	9.9762*E* − 87
Rank	5	2	1	7	6	4	3
*f*_8_							
Mean	0	0	0	0	0	0	0
Rank	1	1	1	1	1	1	1
Std	0	0	0	0	0	0	0
Rank	1	1	1	1	1	1	1
*f*_9_							
Mean	1.1555*E* − 90	2.5372*E* − 78	1.6308*E* − 76	2.6199*E* − 86	1.4655*E* − 89	1.3155*E* − 117	6.1364*E* − 130
Rank	3	6	7	5	4	2	1
Std	2.7315*E* − 90	1.1059*E* − 77	4.7755*E* − 76	7.7290*E* − 86	6.2719*E* − 89	5.7340*E* − 117	2.6737*E* − 129
Rank	3	6	7	5	4	2	1
*f*_10_							
Mean	2.2792*E* − 128	5.5926*E* − 118	9.1955*E* − 124	3.0530*E* − 130	2.8788*E* − 130	6.7603*E* − 161	1.6779*E* − 167
Rank	5	7	6	4	3	2	1
Std	9.7764*E* − 128	2.4326*E* − 117	3.4455*E* − 123	9.2801*E* − 130	1.1346*E* − 129	2.9329*E* − 160	0
Rank	5	7	6	3	4	2	1
*f*_11_							
Mean	−4.1743*E* + 04	−4.1279*E* + 04	−4.1366*E* + 04	−4.1366*E* + 04	−4.1345*E* + 04	−4.1757*E* + 04	−4.1790*E* + 04
Rank	3	6	4	4	5	2	1
Std	4.3502*E* + 02	4.1356*E* + 02	3.5331*E* + 02	4.1470*E* + 02	3.4657*E* + 02	2.6837*E* + 02	1.4566*E* + 02
Rank	7	5	4	6	3	2	1
*f*_12_							
Mean	0	0	0	0	0	0	0
Rank	1	1	1	1	1	1	1
Std	0	0	0	0	0	0	0
Rank	1	1	1	1	1	1	1
*f*_13_							
Mean	1.4998*E* − 32	1.4998*E* − 32	1.4998*E* − 32	1.4998*E* − 32	1.4998*E* − 32	1.4998*E* − 32	1.4998*E* − 32
Rank	1	1	1	1	1	1	1
Std	0	0	0	0	0	0	0
Rank	1	1	1	1	1	1	1

**Table 14:** The mean, standard deviation, and corresponding ranks of best solutions obtained by different optimization strategies of PS-FW for functions *f*_14_ to *f*_22_ (the best ranks are marked in bold).

*f*(*x*)	Strategy-1	Strategy-2	Strategy-3	Strategy-4	Strategy-5	Strategy-6	Strategy-7
*f*_14_							
Mean	6.4751*E* − 275	4.6790*E* − 268	5.0050*E* − 272	1.2035*E* − 283	9.7967*E* − 265	0	0
Rank	3	5	4	2	6	1	1
Std	0	0	0	0	0	0	0
Rank	1	1	1	1	1	1	1
*f*_15_							
Mean	0	0	0	0	0	0	0
Rank	1	1	1	1	1	1	1
Std	0	0	0	0	0	0	0
Rank	1	1	1	1	1	1	1
*f*_16_							
Mean	2.4731*E* − 93	2.5574*E* − 102	1.0668*E* − 102	9.2122*E* − 91	7.8026*E* − 91	2.5290*E* − 114	1.7103*E* − 116
Rank	5	4	3	7	6	2	1
Std	8.4009*E* − 93	1.0215*E* − 101	3.2290*E* − 102	3.7019*E* − 90	3.0225*E* − 90	4.6404*E* − 114	6.2900*E* − 116
Rank	5	4	3	7	6	2	1
*f*_17_							
Mean	0	0	0	0	0	0	0
Rank	1	1	1	1	1	1	1
Std	0	0	0	0	0	0	0
Rank	1	1	1	1	1	1	1
*f*_18_							
Mean	0	0	0	0	0	0	0
Rank	1	1	1	1	1	1	1
Std	0	0	0	0	0	0	0
Rank	1	1	1	1	1	1	1
*f*_19_							
Mean	9.0096*E* − 250	2.3878*E* − 201	1.5857*E* − 189	5.9464*E* − 249	1.5925*E* − 244	0	0
Rank	2	5	6	3	4	1	1
Std	0	0	0	0	0	0	0
Rank	1	1	1	1	1	1	1
*f*_20_							
Mean	1	1	1	1	1	1	1
Rank	1	1	1	1	1	1	1
Std	0	0	0	0	0	0	0
Rank	1	1	1	1	1	1	1
*f*_21_							
Mean	0	0	0	0	0	0	0
Rank	1	1	1	1	1	1	1
Std	0	0	0	0	0	0	0
Rank	1	1	1	1	1	1	1
*f*_22_							
Mean	4.9253*E* − 273	8.5544*E* − 231	1.4963*E* − 229	3.8782*E* − 275	4.3846*E* − 276	0	0
Rank	4	5	6	3	2	1	1
Std	0	0	0	0	0	0	0
Rank	1	1	1	1	1	1	1

**Table 15:** The results of Friedman test for the different strategies of PS-FW over the mean and standard deviation of optimal solutions based on Tables 13 and 14 (the best ranks are marked in bold).

	Mean	Std
Results		
*N*	22	22
Chi-square	40.23	22.38
*p* value	4.10*E* − 07	1.03*E* − 03
Friedman ranks of algorithms		
Strategy-1	3.91	4.14
Strategy-2	4.75	4.25
Strategy-3	4.52	4.23
Strategy-4	4.5	4.52
Strategy-5	4.64	4.27
Strategy-6	2.95	3.41
Strategy-7	2.73	3.18
